# Evaluation of an experiential clinical learning option during pandemic teaching suspensions

**DOI:** 10.1186/s12909-022-03530-4

**Published:** 2022-06-17

**Authors:** Jules Canfield, Ve Truong, Agata Bereznicka, Karsten Lunze

**Affiliations:** 1 Clinical Addiction Research and Education Unit, Section of General Internal Medicine, Boston Medical Center, 801 Massachusetts Avenue, Room 2045, Boston, MA 02118 USA; 2 Boston University School of Medicine, Boston, MA 02118 USA

**Keywords:** Addiction medicine, Medical education, Remote learning, COVID-19

## Abstract

**Background:**

As students’ direct patient contact was suspended because of COVID-19-related restrictions, we revised our clinical addiction medicine curriculum for students to learn about the different multidisciplinary clinical models delivered at our hospital and in community settings. Our aim was to provide an overview of clinical modalities and familiarize learners with clinician and patient experiential perspectives.

**Methods:**

We implemented a multi-pronged approach, offering an overview of clinical care programs through remote panels involving care providers at the clinics where students had previously been scheduled for in-person rotations. This included inpatient and office-based addiction services, addiction treatment program for adolescents and young adults, integrated addiction care and HIV primary care clinic, and opioid use urgent care clinic. Beyond having them join outpatient telehealth clinic visits, students also participated in an online panel involving patients in recovery to gain familiarity with their care perspectives; and joined a panel with recovery coaches to get further insights into patient challenges in clinical settings. Students further participated in remote opioid treatment trainings and observed clinical rounds of inpatient addiction consults and adolescent clinic team meetings.

**Results:**

With this revised curriculum, students learned about the variety of clinical modalities at the height of our hospital’s COVID-19 pandemic burden. The evaluation suggested that students appreciated the authenticity of accounts from patients and providers about their challenges and satisfaction related to clinical care. While in a remote learning setting, students overall wished for more personal interaction with patients and providers. They also noted a lack of group cohesion and connection that they felt would otherwise have been met in an in-person program.

**Conclusions:**

Remote learning allowed our program to connect trainees to the multidisciplinary field of addiction medicine despite the COVID-19 pandemic. In future program iterations, we will consider hybrid formats of in-person learning experiences with direct patient and faculty contact where possible, combined with online provider and patient panels possibly, in addition to virtual breakout formats to facilitate more personal student-patient and student-faculty interactions.

**Supplementary Information:**

The online version contains supplementary material available at 10.1186/s12909-022-03530-4.

## Background

The COVID-19 pandemic has led to numerous changes in academic, research, and learning environments over the past two years. While virtual modalities have been commonplace in the United States since before the pandemic, there has been a significant shift from in-person to full-remote teaching due to social distancing requirements [1]. Virtual learning provides an opportunity to continue the education process, but has created challenges in the implementation of research activities as well as limited beneficial face-to-face social interaction.

While substance use disorders are a major cause of mortality [2], adequate addiction medicine and research education is lacking at the medical school level [3]. To address this issue, we have delivered a structured student clinical addiction medicine program at our academic teaching hospital. This student research program traditionally offers student rotations in inpatient, outpatient and community settings to complement mentored research and a seminar series. Clinical placements are important for students as they transition from preclinical to clinical learning. The COVID-19 pandemic and related lockdowns required the cancellation or modification of most clinical rotations [4].

At our institution, suspension of all clinical rotations due to the COVID‐19 pandemic required a rapid transition to physically distanced education and precepting. As was the case for many clinical training programs, we had students join addiction treatment outpatient clinic visits via telehealth. Such telehealth encounter require establishing processes to obtain patient consent, ensure professionalism and maintain confidentiality [5]. These are standard aspects of student encounters with patients, but they needed some advance preparation with preceptors before students were prepared to connect with patients. Following the initial shift to “teleprecepting” medical students, clinic preceptors could follow similar principles as in-person precepting [6].

To meet educational needs for a diverse student population, we also offered weekly 60–90 min online seminars over the course of six weeks. These seminars provided a didactic introduction to addiction medicine and to the management of various substance use disorders. Given the video-conferencing capacity established at most educational facilities, this virtual teaching modality required only minor changes to previous in-person teaching formats.

Virtual learning formats thus provide valuable opportunities for distant learning under pandemic conditions [7, 8]. Accordingly, due to the COVID-19 pandemic, several medical education programs shifted their teaching techniques [9, 10], However, remote formats alone could not acquaint learners with different care models nor do they familiarize them with diverse clinician and patient experiential perspectives: telehealth clinic encounters and online seminars alone provide a limited experience to learners [11–13]. Few programs addressed both clinical observations and research skills among first year medical students [14–16].

For students to learn about the various addiction treatment service models at our hospital and the multidisciplinary care modalities various providers deliver in these respective settings, we complemented telehealth learning with remote clinical observations (i.e. Doximity), as well as virtual panels of multiple professionals and patients (i.e. Zoom). Our goal was to describe a new program, conducted under pandemic conditions for students to become familiar with clinician and patient perspectives of different care models and to gain exposure to patients with lived addiction experiences.

## Methods

In order to provide an in-depth overview of the clinical addiction care programs in the hospital, we held weekly, 50-min teleconferencing panels on Zoom with two providers at each of the clinics where students were originally scheduled to observe in. Each virtual panel connected students with a physician and other providers such as nurses, counselors, and social workers. We offered provider panels at each of the following sites: inpatient addiction consult service, addiction treatment program for adolescents and young adults, integrated addiction care and HIV primary care program, and opioid use urgent care center referring patients with substance use disorders to a comprehensive care network of inpatient and outpatient services. In these panels, providers discussed their clinical training and practices, as well as the respective clinic care model, including diagnostic and therapeutic modalities.

Additionally, in order for students to interact with patients and gain familiarity with their care perspectives, we held a 50-min, provider-moderated online panel with two patients in recovery from substance use disorders. Physicians of these individuals with lived experiences approached them specifically to share their experiences and perspectives as patients at specific clinics. These individuals were selected based on their insightfulness and comfort discussing their experiences with substance use. We scheduled those who agreed to engage in a panel discussion with the students, which was facilitated by their physicians. We complemented this with a panel featuring recovery coaches, i.e., individuals employed by clinics who provide patient navigation and support. As this health care cadre has a distinct educational background and function in patient care, they provided additional insights into patient experiences and challenges in clinical settings.

Because students’ in-person patient encounters were suspended, we also arranged for students to engenge via phone or through teleconferencing in telehealth outpatient clinic sessions, opioid treatment trainings for health centers, observations of clinical rounds of an inpatient addiction consult and of the adolescent clinic interdisciplinary team discussions of current patients.

To evaluate our program, we administered surveys that included Likert-scale quantitative questions about students’ satisfaction with the various aspects of the program; and structured, open-ended qualitative question exploring students’ learning experiences and their perceptions of having their learning goals for the program fulfilled. We collected data via REDCap and administered 1) a pre-program baseline survey and post-program evaluation, and 2) patient and provider panel evaluations after each session.

## Results

Of the 11 students who participated in the program during the COVID‐19 pandemic (Table 1), less than one in three had ever worked in a substance use treatment setting (27%) or visited either a 12-step program or a substance use treatment facility (30%) before starting the program. Almost half (46%) had previously trained at a non-US institution. In their program evaluations, all students indicated enjoyment in learning about substance use and interest in conducting substance use-related research in their future career at baseline. From program start to program completion, participants reported increased interest in conducting addiction research in a future career, and to be more likely to specialize in substance use clinical care or research after completing their education.

**Table 1:** 2020 Sumer research program participant demographics (*n* = 11) & program evaluation data

	**Baseline**	**After Program Completion**
Female	91%	
Race:		
Asian	55%	
White	45%	
Student status:		
Medical	82%	
Undergraduate	9%	
Other	9%	
Ever attended a non-U.S. educational institution	46%	
Ever worked or volunteered in a substance use treatment setting	27%	
Ever visited either a 12-step program or a substance use treatment facility	30%	
Enjoy learning about substance use *(Scale of Least* = *1 – Most* = *5)*	4.45	
How interested are you in conducting substance use-related research in your future career? *(Scale of Least* = *1 – Most* = *5)*	4.36	4.5
After completing medical school/other educational training, how likely are you to specialize in substance use, clinical or research? *(Scale of Least* = *1 – Most* = *5)*	2.73	3.6
Overall, how useful was the addiction medicine observation series to you? *(Scale of Least* = *1 – Most* = *5)*		4.6
How clear were directions and scheduling for your observations? *(Scale of Least* = *1 – Most* = *5)*		5
How accommodating was the mentor to your observation experience? *(Scale of Least* = *1 – Most* = *5)*		5
Would recommend this panel for next year: *(Yes)*		
Urgent care center for patients with substance use disorders		64%
Clinic for adolescents and emerging adults		64%
Inpatient addiction Consult Service		73%
Recovery Coaches		73%
Primary care clinic for people with HIV and substance use disorders		73%
Individuals in Recovery		73%
How useful is hearing patient perspectives to you? *(Scale of Least* = *1 – Most* = *5)*		5

Almost all students commented on the impact of the patient and provider panels and how much they had learned about clinician and patient experiences with addiction treatment. All students who attended the provider panels recommended hosting them again, and all who attended the patient panel expressed that they found it useful to hear patient perspectives.

Some students noted that they developed an understanding about the intersection of stigma and addiction, impact of trauma on seeking treatment for substance use disorders, and multi- and inter-disciplinary approaches within the field of addiction. Almost all students stressed the importance of learning about different treatment programs and modalities, and how the various clinic models related to each other. All students appreciated the authenticity of accounts from patients and providers about the challenges and rewards related to clinical care. Hearing from individuals in recovery, several felt that patients on the panels had “incredibly unique or useful perspectives” and insights to share. One student stated, “The patients inspired me with their stories. Their work was a great illustration of patient involvement in advocacy and teaching.”

Overall, all students expressed that the virtual format worked effectively given the circumstances, with several stating that it provided them with additional flexibility and the ability to more easily attend program activities. However, several students added that the remote format felt less personal and there was a lack of group or cohort cohesion. Some students also stated that they would have liked longer sessions for the patient and provider panels so that they could more engage in discussions.

## Discussion

The COVID-19 pandemic required major changes in clinical student trainings [17]. We revised our curriculum to include virtual provider and patient panels, complementing online and telehealth clinic opportunities. Experiential learning is particularly important for medical students to develop clinical skills and empathetic approaches to patient care [18], especially to serve those with substance use disorders [19]. The revised format allowed students to gain exposure in the variety of clinical addiction programs while our hospital was burdened with the COVID-19 pandemic. Students became exposed to the experiences of individuals in recovery and the daily experiences of providers who work in the various settings in clinical and community addiction services.

Other programs have used role-play or simulation scenarios to provide remote experiences of clinical rotations [20, 21].These formats can complement and enhance our virtual clinical engagement through provider and patient panels.

The patient panel gave individuals a voice to speak about their experiences, challenges and successes of being in recovery. The recovery coaches also added important insight on caring for people living with substance use disorders from both a personal and professional level [22]. While in response to the COVID-19 pandemic many medical schools modified their clinical placements for medical students to digital formats without exposure to patients [23], our revised curriculum was able to maintain interaction with “live” patients. As we continued to offer didactic sessions as online seminars, students particularly valued the authenticity of accounts from patients and providers about the challenges and rewards related to clinical care.

In the remote formats, students are limited in their ability to learn procedural tasks, and individual interactions with patients are also limited. Near-peer-teaching and peer learning has become increasingly common during the pandemic to address this deficit [24]. Under pandemic conditions, hospitals can provide studens with new roles in patient care, adding to their learning opportunities [25]. Students could be offered modified in-person clinical rotations with pandemic precautions to complement the remote experiences we implemented [26]. These teaching modalities should promote team-based and problem-based learning, possibly in a hybrid approach of in-person and remote learning [27].

### Limitations

Given the small sample size, we were unable to validate our questions’ reliability or internal consistency. For the same reasons, this data’s external validity is very limited. Qualitative data are not generalizable beyond the sample and thus primarily reflect the perspectives of this group.

## Conclusion

The educational disruptions caused by the COVID-19 pandemic prompted us to leverage the potential of remote learning, with valuable guidance gained through experiences made worldwide [28]. Strengths of this virtual approach include the ability to accommodate a larger number of learners, as well as to include students residing further away from our hospital, including learners in international settings. As the COVID-19 pandemic transitions to endemic and in-person learning is safe for our participants, we aim to return to a predominately in-person program, with the potential for a hybrid model so that participants outside of the Boston area can be accommodated. In future iterations of our summer research program we plan to continue our highly-rated patient panel as well as our recovery coach provider panel, as these rich insights are unlikely to be gained by our participants solely through the in-person clinical observations.

## Supplementary Information

**Additional file 1. **Medical Student Summer Research Program Questionnaires.

## Data Availability

The datasets used and/or analysed during the current study are available from the corresponding author on reasonable request.
