# Supplementary material for: Evaluation of an experiential clinical learning option during pandemic teaching suspensions
Source: BMC Med Educ. 2022 Jun 17;22:471. doi: 10.1186/s12909-022-03530-4 (PMC9205138; doi:10.1186/s12909-022-03530-4)
Supplement: Supplementary file 1 — Additional file 1. Medical Student Summer Research Program Questionnaires. [file 12909_2022_3530_MOESM1_ESM.pdf]

2020 Baseline Assessment, MSSRP

Please complete the survey below. NOTE: Allow yourself at least 15 minutes to complete this survey. In the following questions, the terms: substance use includes drugs and alcohol, not tobacco products. Drugs does not include alcohol or tobacco products.

Your Background

1) Have you ever worked or volunteered in a substance use treatment setting for a month or more?

◯ Yes
◯ No

2) Have you ever visited the following in a professional context?

◯ Yes, a 12-step program (AA or NA)
◯ Yes, a substance use treatment facility only (e.g. detox, methadone-maintenance)
◯ Yes, both a 12-step program and a substance use treatment facility
◯ No, I have not visited either a 12-step program or a substance use treatment facility

3) Over the past year, how many hours have you participated in each of the following when substance use was the topic? (Please use numbers, e.g. "2" rather than "two." Also, indicate "0" if you have not participated in the activity.)

...conferences (e.g., journal club, grand rounds, morning report, attending rounds, M&M conferences) ________________________

...educational programs at your institution (e.g., courses, online courses, seminars, lectures) ________________________

...educational programs external to your institution (e.g., courses, seminars, lectures, conferences) ________________________

Your Background in Addiction Medicine and Research

Which of the following journals have you read in the past year? Check all that apply.

☐ Addiction
☐ Drug and Alcohol Dependence
☐ Journal of Studies on Alcohol and Drugs
☐ Other

If other, please describe ________________________

Your Education Experience

Which substance use (SU) topics were covered in your educational experience? Check all that apply no matter what the venue.

☐ Did not learn about substance use in the past 6 months
☐ Neurobiology of substance use
☐ Epidemiology of substance use
☐ Screening and assessment
☐ Stages of behavioral change
☐ Brief intervention counseling strategies
☐ Evidence-based SU treatment approaches
☐ Interplay between pain and addiction
☐ Comorbidities with SU (e.g., hepatitis and SU)
☐ Other substance use topics

Please specify "other" in the box below.

____

Do not enjoy at all | Enjoy very much

How much do you enjoy learning about substance use?	○	○	○	○	○
	Not at all confident				Very confident
Overall, how confident are you about your knowledge of substance use?	○	○	○	○	○
	Not included at all				Included a great deal
Overall, to what extent is training about substance use included in your education?	○	○	○	○	○

Your Future Plans

	Not at all interested				Very interested
How interested are you in conducting substance use-related research in your future career?	○	○	○	○	○

After completing medical school/other educational training, how likely are you to specialize in substance use, clinical or research?

○ Not at all ○ ○ Somewhat ○ ○ Very much

What experience have you had, if any, in the following tasks?

a) Had this experience

	Yes	No
Grant preparation and submission	○	○
Submission of a proposal to an IRB	○	○
Research design	○	○
Data collection	○	○
Data analysis	○	○
Writing up results (e.g., manuscript presentation, poster, or oral presentation)	○	○
Working with someone you consider to be a research mentor on any or all of the above	○	○

b) Addressed issues related to substance use?

	Yes	No
Grant preparation and submission	○	○
Submission of a proposal to an IRB	○	○
Research design	○	○
Data collection	○	○
Data analysis	○	○
Writing up results (e.g., manuscript presentation, poster, or oral presentation)	○	○
Working with someone you consider to be a research mentor on any or all of the above	○	○

How confident are you in performing the following?

	Not at all confident	2	3	4	Very confident
Working with a mentor	○	○	○	○	○
Developing a research question based on available or new data	○	○	○	○	○
Describing different study designs and their pros and cons for answering different kinds of questions	○	○	○	○	○
Preparing an IRB proposal for a research project	○	○	○	○	○
Conducting research studies with ethical conduct and human participation protections in mind	○	○	○	○	○
Conducting a literature search or review relevant to your research project	○	○	○	○	○
Collecting data, whether that be from appropriate databases, research participants, or other relevant sources	○	○	○	○	○
Entering and managing data, including quality control	○	○	○	○	○
Analyzing data, including using statistical software or analytic procedures	○	○	○	○	○

Working on a research team (e.g., with research assistants, statistician, project manager)	◯	◯	◯	◯	◯
Interpreting and writing up a discussion of your results, whether as a poster or publication	◯	◯	◯	◯	◯
Submitting research for publication in journals	◯	◯	◯	◯	◯
Submitting research for presentation at scientific conferences	◯	◯	◯	◯	◯

Demographic Information

34) Have you or are you currently attending a non-U.S. (or foreign) educational institution?

◯ Yes
◯ No

Gender:

◯ Female
◯ Male
◯ Prefer to self describe

If you selected "prefer to self describe," please specify:

____

Ethnicity:

◯ Hispanic or Latino
◯ Not Hispanic or Latino

Race:
Check all that apply.

☐ American Indian/Alaskan Native
☐ Asian
☐ Native Hawaiian/Other Pacific Islander
☐ Black/African American
☐ White
☐ Other (specify other in box below)

Please specify "other" in the box below.

____

Sexuality:

◯ Heterosexual
◯ Bisexual
◯ Homosexual
◯ Other
◯ Prefer not to disclose

Please specify "other" in the box below.	__________________________________
Do you have a disability?	◯ Yes<br>◯ No<br>◯ Prefer not to disclose
Please indicate your current status as a student:	◯ Medical student<br>◯ Undergraduate student<br>◯ Graduate student<br>◯ Other
Please specify "other" in the box below:	__________________________________
Please list your current institution in the box below:	__________________________________
Please provide feedback about the survey or any additional comments.	

Evaluation for Addiction Medicine Observations & CARE Summer Project

Please complete the survey below.

Thank you!

1) I am a:
- ◯ Medical Student
- ◯ Undergraduate Student
- ◯ Other

For each observation you attended, on a scale of 1-5 (with 5 being very accommodating), how accommodating was the mentor (host) to your observation experience?

		1 (Not at all)	2	3	4	5 (Very)	N/A (Did not observe)
2)	Faculty Mentor or Designated Provider	◯	◯	◯	◯	◯	◯
3)	Addiction Consult Service Rounds	◯	◯	◯	◯	◯	◯
4)	ECHO	◯	◯	◯	◯	◯	◯
5)	CATALYST Team Meeting	◯	◯	◯	◯	◯	◯
6)	SCOPE of Pain Training(s)	◯	◯	◯	◯	◯	◯
7)	OBAT Training (s)	◯	◯	◯	◯	◯	◯

		1 (Least useful)	2	3	4	5 (Most useful)	N/A (did not observe)
8)	Overall, how useful was the addiction medicine observation series to you?	◯	◯	◯	◯	◯	◯

		1 (Not at all useful)	2	3	4	5 (Very useful)	N/A (Did not observe)
9)	How clear were directions and scheduling for your observations?	◯	◯	◯	◯	◯	◯

10) Do you have any suggestions or comments for directions and scheduling?

____

11) Do you have any comments or suggestions for the mentors (hosts)?

____

12) Do you have any other general suggestions for addiction medicine observations?

____

13) What were your learning goals this summer?

____

14) Were these goals met or unmet, and in what way?

____

15) What was the MOST useful or valuable experience during your summer project?

____

16) What was the LEAST useful or valuable experience during your summer project?

____

How confident are you in performing the following?

	Not at all confident	2	3	4	Very confident
17) Working with a mentor	○	○	○	○	○
18) Developing a research question based on available or new data	○	○	○	○	○
19) Describing different study designs and their pros and cons for answering different kinds of questions	○	○	○	○	○
20) Preparing an IRB proposal for a research project	○	○	○	○	○
21) Conducting research studies with ethical conduct and human participation protections in mind	○	○	○	○	○
22) Conducting a literature search or review relevant to your research project	○	○	○	○	○
23) Collecting data, whether that be from appropriate databases, research participants, or other relevant sources	○	○	○	○	○
24) Entering and managing data, including quality control	○	○	○	○	○
25) Analyzing data, including using statistical software or analytic procedures	○	○	○	○	○
26) Working on a research team (e.g., with research assistants, statistician, project manager)	○	○	○	○	○
27) Interpreting and writing up a discussion of your results, whether as a poster or publication Submitting research for publication in journals	○	○	○	○	○
28)					

Submitting research for presentation at scientific conferences ○ ○ ○ ○ ○

29) Overall, how confident are you about your knowledge of substance use?

____

30) How much do you enjoy learning about substance use?

____

31) How interested are you in conducting substance use-related research in your future career?
○ Not at all interested ○ 2
○ 3 ○ 4 ○ Very interested

32) After completing medical school/other educational training, how likely are you to specialize in substance use, clinical or research?
○ Not at all ○ 2 ○ Somewhat
○ 4 ○ Very much

33) What was your overall experience with the remote/virtual format? What worked and did not work?

____

34) Do you have any suggestions for improvement?

____

2020 Patient Panel

Please complete the survey below.

Thank you!

---

1) What were the most useful information or insights you learned at this panel? ______________________________

2) What could be improved or removed from the panel? ______________________________

3) How useful is hearing patient perspectives to you?
   - ◯ Not at all
   - ◯
   - ◯ Somewhat
   - ◯
   - ◯ Very much

4) Would you recommend this panel for next year? Why or why not? ______________________________

5) Other comments or suggestions for improving the panel series: ______________________________

2020 Provider Panel

Please complete the survey below.

Thank you!

1) What were the most useful information or insights you learned at the panel?

____

2) What could be improved or removed from the panel?

____

3) Do you feel the readings assigned beforehand prepared you for this session?

- ◯ Yes
- ◯ No
- ◯ I did not read the articles beforehand
- ◯ N/A - there were no articles assigned beforehand

4) Please explain further:

____

5) Would you recommend this panel for next year? Why or why not?

____

6) Other comments or suggestions for improving the panel series:

____
